# Biological activities of frankincense essential oil in human dermal fibroblasts

**DOI:** 10.1016/j.biopen.2017.01.003

**Published:** 2017-02-03

**Authors:** Xuesheng Han, Damian Rodriguez, Tory L. Parker

**Affiliations:** dōTERRA International, LLC, 389 S. 1300 W, Pleasant Grove, UT 84062, USA

**Keywords:** Inflammation, Immune response, Tissue remodeling, Alpha-pinene, Anti-proliferation, Skin health

## Abstract

Although frankincense essential oil (FREO) has become increasingly popular in skin care, research on its biological activities in human skin cells is scarce, if not completely absent. In the current study, we explored the biological activities of FREO in pre-inflamed human dermal fibroblasts by analyzing the levels of 17 important protein biomarkers pertinent to inflammation and tissue remodeling. FREO exhibited robust anti-proliferative activity in these skin cells. It also significantly inhibited collagen III, interferon gamma-induced protein 10, and intracellular cell adhesion molecule 1. We also studied its effect in regulating genome-wide gene expression. FREO robustly modulated global gene expression. Furthermore, Ingenuity^®^ Pathway Analysis showed that FREO affected many important signaling pathways that are closely related to inflammation, immune response, and tissue remodeling. This study provides the first evidence of the biological activities of FREO in human dermal fibroblasts. Consistent with existing studies in other models, the current study suggests that FREO possesses promising potential to modulate the biological processes of inflammation and tissue remodeling in human skin. Further research into the biological mechanisms of action of FREO and its major active components is recommended.

## Highlights

• Frankincense essential oil (FREO) was anti-proliferative to human dermal fibroblasts.
• FREO significantly inhibited the production of collagen III, IP-10, and ICAM-1.
• FREO robustly modulated genome-wide gene expression.
• FREO affected signaling pathways related to inflammation and tissue remodeling.

## 1 Introduction

Frankincense is a resin obtained from trees of the genus *Boswellia.* Historically, frankincense whole resin, its extract, and essential oil have been extensively used for a number of health purposes in Chinese and Ayurvedic medicine. FREO^1^ has been traditionally used for its anti-inflammatory property. Recently, FREO has become increasingly popular for promoting skin health. However, a literature search showed no published study of the biological activities of FREO in human skin cells.

In this study, we explored the biological activities of a commercially available FREO in human dermal fibroblasts *in vitro.* We first studied the effect of FREO on the levels of 17 important biomarkers related to inflammation, immune response, and tissue remodeling in the skin cells. Then, we studied the effect of FREO on the expression levels of 21,224 genes, using genome-wide analysis of the same cells. The results showed that FREO was biologically active and significantly affected expression of these biomarkers and genes.

## 2 Materials and methods

All experiments were conducted in a BioMAP HDF3CGF system, a cell culture of human dermal fibroblasts that is designed to model chronic inflammation and fibrosis in a robust and reproducible way. The system consists of three components: a cell type, stimuli to create the disease environment, and set of biomarker (protein) readouts to examine how treatments affect that disease environment [1].

### 2.1 Cell cultures

Primary human neonatal fibroblasts (HDFn) were obtained as previously described [2]. HDFn were plated in low serum conditions, 24-h before stimulation with cytokines. Cell culture and stimulation conditions for HDF3CGF assays have been described in detail elsewhere, and were performed in a 96-well format [2], [3].

### 2.2 Protein-based readouts

Direct ELISA was used to measure the biomarker levels of cell-associated and cell membrane targets. Soluble factors from supernatants were quantified using HTRF^®^ detection, bead-based multiplex immunoassay, or capture ELISA. Overt adverse effects of the test agents on cell proliferation and viability (i.e., cytotoxicity) were measured using SRB^2^ assay. For proliferation assays, cells were cultured and then assayed after 72 h, which was optimized for the HDF3CGF system. Detailed information has been described elsewhere [2]. Measurements were performed in triplicate wells. See Table S1 in Supplementary Materials for a glossary of the biomarkers used in this study.

### 2.3 RNA isolation

Total RNA was isolated from cell lysates using the Zymo *Quick-RNA*™ MiniPrep kit (Zymo Research Corporation, Irvine, CA), according to manufacturer's instructions. RNA concentration was determined using NanoDrop ND-2000 (Thermo Fisher Scientific). RNA quality was assessed with a Bioanalyzer 2100 (Agilent Technologies, Santa Clara, CA) and an Agilent RNA 6000 Nano Kit. All samples had an A260/A280 ratio between 1.9 and 2.1, and an RNA Integrity Number score greater than 8.0.

### 2.4 Microarray analysis for genome-wide gene expression

A 0.003% (v/v) concentration of FREO was tested for its effect on expression of 21,224 genes in the HDF3CGF system after 24-h treatment. Samples for microarray analysis were processed by Asuragen, Inc. (Austin, TX), according to the company's standard operating procedures. Biotin-labeled cRNA was prepared from 200 ng of total RNA with an Illumina^®^ TotalPrep™ RNA Amplification kit (Thermo Fisher Scientific) and one round of amplification. The cRNA yields were quantified via UV spectroscopy, and the distribution of transcript sizes was assessed using the Agilent Bioanalyzer 2100. Labeled cRNA (750 ng) was used to probe Illumina Human HT-12 v4 Expression BeadChips (Illumina, Inc., San Diego, CA). Hybridizing, washing, staining with streptavidin-conjugated Cyanine-3, and scanning of the Illumina arrays was performed according to the manufacturer's instructions. Illumina BeadScan software was used to produce the data files for each array; raw data were extracted using Illumina BeadStudio software.

Raw data were uploaded into R [3] and analyzed for quality-control metrics using the *beadarray* package [4]. Data were normalized using quantile normalization [5], then re-annotated and filtered to remove probes that were non-specific or mapped to intronic or intragenic regions [6]. The remaining probe sets comprised the data set for the remainder of the analysis. Fold-change expression for each value was calculated as the log_2_ ratio of FREO to vehicle control. These fold-change values were uploaded to Ingenuity^®^ Pathway Analysis (IPA^®^,^3^ QIAGEN, Redwood City, CA, www.qiagen.com/ingenuity) to generate the network and pathway analyses.

### 2.5 Reagents

FREO (dōTERRA International LLC, Pleasant Grove, UT, USA) was diluted in DMSO to 8X the specified concentrations (final DMSO concentration in culture media was no more than 0.1% (v/v)); 25 μL of each 8X solution was added to the cell culture to a final volume of 200 μL. DMSO (0.1% (v/v)) served as the vehicle control. Gas chromatography–mass spectrometry analysis of FREO indicated that its major chemical constitutes (i.e., >5%) were alpha-pinene (57%), limonene (8%), and caprylyl acetate (7%).

## 3 Results

### 3.1 Bioactivity profile of FREO in pre-inflamed human dermal fibroblasts

**Fig. 1:**
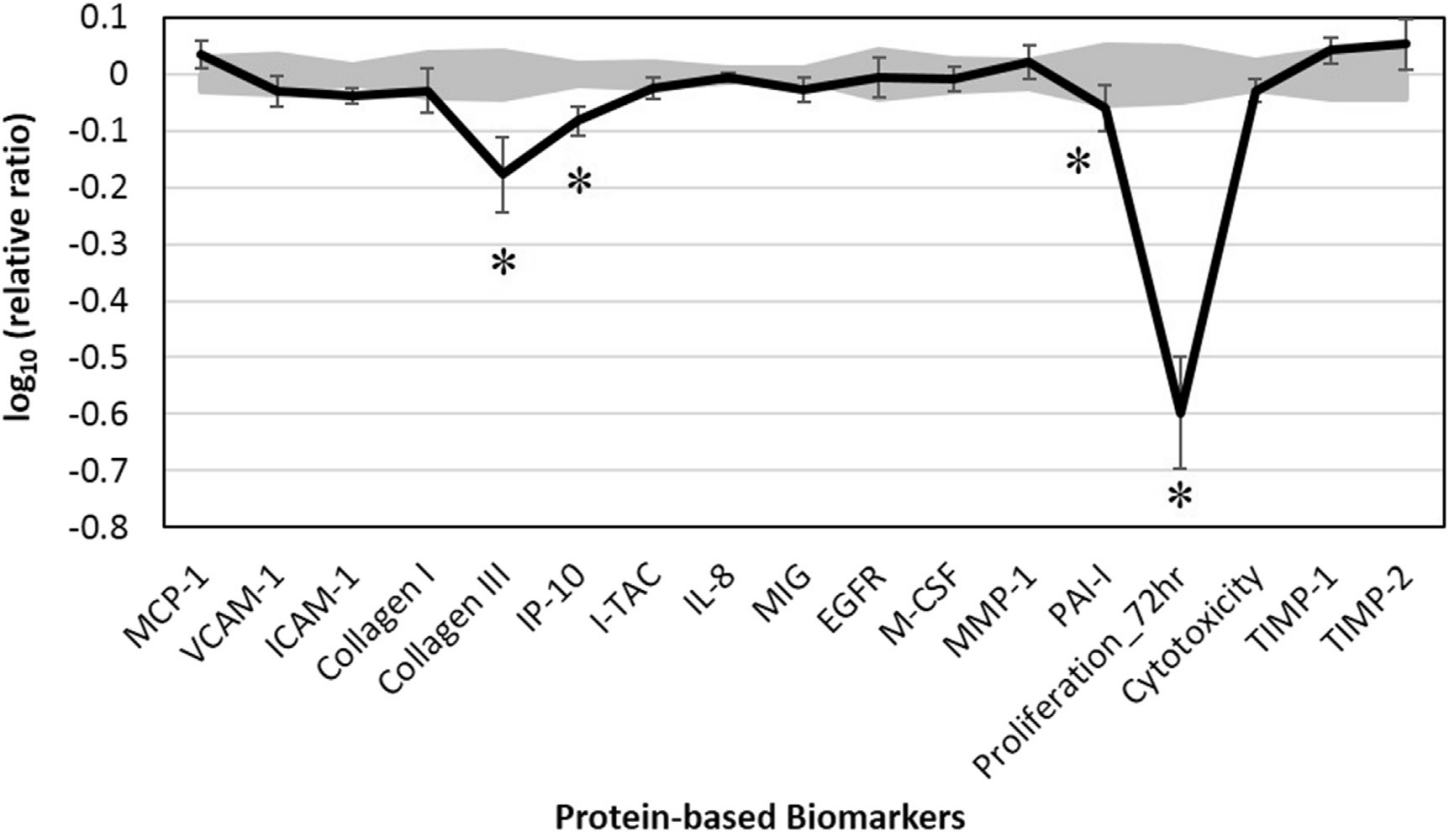
The bioactivity profile of FREO (0.003% (v/v) in DMSO) in BioMAP System HDF3CGF. X-axis denotes protein-based biomarker readouts. Y-axis denotes the relative expression levels of biomarkers compared to vehicle control values, in log form. Vehicle control values are shaded in gray, denoting the 95% confidence level. A * indicates a biomarker designated with “key activity,” i.e., biomarker values (presented as mean ± SD, from three measurements) were significantly different (p < 0.05) from vehicle controls, with an effect size of at least 10% (more than 0.05 log ratio units).

Four different concentrations (0.003, 0.001, 0.00033, and 0.00011% (v/v)) of FREO were initially tested for biological activity in the dermal fibroblasts. None of the four concentrations was overtly cytotoxic, and therefore, the highest concentration (i.e., 0.003%) was analyzed further. FREO showed significant anti-proliferative activity in dermal fibroblasts. Biomarkers were designated if FREO values were significantly different (p < 0.05) from vehicle controls, with an effect size of at least 10% (more than 0.05 log ratio units) (Fig. 1). The level of a tissue remodeling biomarker, collagen III, decreased in response to FREO. FREO significantly reduced levels of interferon gamma-induced protein 10 (IP-10^4^) and intracellular cell adhesion molecule 1 (ICAM-1^5^), both important inflammatory biomarkers. FREO also slightly lowered the levels of PAI-I, serine proteinase inhibitor and inhibitor of tissue plasminogen activator (tPA) and urokinase (uPA), which is involved in tissue remodeling.

### 3.2 Effects of FREO on gene expression: a genome-wide total RNA expression assay

**Fig. 2:**
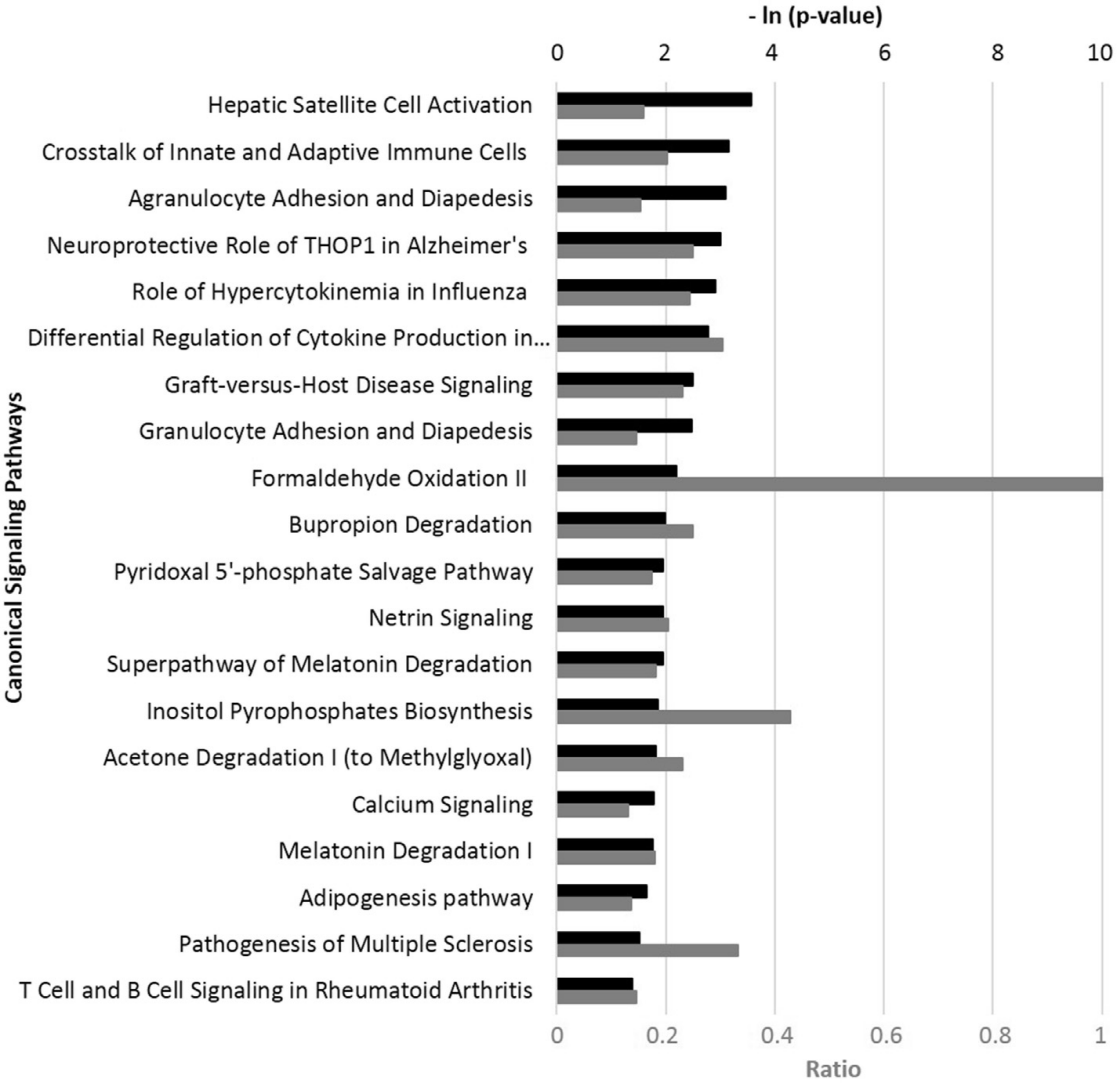
Top 20 canonical pathways matching FREO's bioactivity profile of gene expression in the HDF3CGF system, produced via Ingenuity^®^ Pathway Analysis (IPA^®^, QIAGEN, www.qiagen.com/ingenuity). Each p-value is calculated with the right-tailed Fisher's Exact Test. The p-value measures the likelihood that the observed association between a specific pathway and the dataset is due to random chance. The smaller p value (the bigger – ln (p-value), indicated by the black bars) the pathway has, the more significantly it matches with the bioactivity of FREO. A ratio, indicated by each gray bar, is calculated by taking the number of genes from the FREO dataset that participate in a canonical pathway, and dividing it by the total number of genes in that pathway.

To further explore the effect of 0.003% (v/v) FREO on human skin cells, we analyzed its effect on the RNA expression of 21,224 genes. The results show a robust effect of FREO on regulating human genes, with many genes being upregulated and many others being downregulated. Among the top 83 regulated genes (absolute value of the fold-change ratio of gene expression over vehicle control ≥ 1.5) by FREO, 42 were upregulated, and 41 were downregulated (Table S2). IPA analysis showed that the bioactivity of FREO significantly overlapped with many canonical pathways (Fig. 2) from the literature-validated database (IPA^®^, QIAGEN, Redwood City, CA, www.qiagen.com/ingenuity). Many of these signaling pathways are closely related to the biological processes of inflammation, immune response, and tissue remodeling in human cells. See Supplementary Materials for more details.

## 4 Discussion

### 4.1 The anti-inflammatory and immune-modulating properties of FREO

Inflammation is a protective response that involves immune cells, blood vessels, and molecular mediators. The purpose of inflammation is to eliminate the initial cause of cell injury, remove necrotic cells and tissues damaged from the injury and inflammatory process, and initiate tissue repair. Chronic inflammation may lead to a variety of diseases, such as hay fever, periodontitis, atherosclerosis, rheumatoid arthritis, and cancer [7], [8].

FREO significantly reduced the levels of IP-10 and ICAM-1, important pro-inflammatory biomarkers, suggesting its anti-inflammatory potential. Alpha-pinene, the top constituent of FREO, is widely recognized as the major anti-inflammatory component of FREO. Alpha-pinene showed anti-inflammatory properties in human peripheral blood mononuclear cells and mouse macrophages through inhibition of tumor necrosis factor-α, interleukin-1β, nitric oxide, and mitogen activated protein kinases [9]. An *in vitro* study [10] showed that isolated alpha-pinene had the ability to reduce the expression of pro-inflammatory cytokines. Another study [11] found that alpha-pinene inhibited the nuclear translocation of NF-kB induced by lipopolysaccharide in THP-1 cells, explaining its benefits in the treatment of upper and lower airway diseases.

Recent research has also provided evidence that alpha-pinene has some immune-enhancing properties, particularly regarding enhanced T-cell activity. In two related studies, the effects on human immune function of essential oils from trees were investigated [12], [13]. In both studies, it was found that exposure to alpha-pinene increased T-cell activity and decreased stress hormone levels. Consistent with these studies, microarray results of current study showed that FREO affected some important inflammation- and immune-related signaling pathways. Gene expression of many cytokines and other important players in inflammation and immune responses was significantly inhibited in pre-stimulated, inflamed skin cells, indicating that FREO has potential immune modulating properties.

### 4.2 Potential roles of FREO in the wound healing process

Wound healing is an intricate process, where the skin or other body tissue repairs itself after injury. This process is composed of several phases: blood clotting (hemostasis), inflammation, growth of new tissue (proliferation), and remodeling of tissue (maturation) [14]. The wound healing process is not only complex, but also fragile, and it is susceptible to disruption, leading to the formation of non-healing, chronic wounds [15]. Collagen III is secreted by fibroblasts during the wound remodeling or repairing process, prior to the deposition of collagen I. FREO dramatically lowered the level of collagen III, and therefore, it would likely improve healing by reducing the chance of scar formation or wound persistence. Additionally, the robust, anti-proliferative activity of FREO in skin cells could also contribute to better wound healing.

The significant, anti-proliferative activity of FREO observed in this study may have important implications in skin and other cells. An *in vitro* study showed that FREO induced cell death in J82 bladder cancer cells via NRF-2-mediated oxidative stress [16]. FREO and/or its top constituent, alpha-pinene, has also been shown to be anti-proliferative and pro-apoptotic toward several other types of cancer cells [17], [18], [19], [20], [21]. Further research into FREO's activities in cancer cells will better our understanding of its biological mechanism of action.

## 5 Conclusions

To our knowledge, this was the first study of the biological activities of FREO in human dermal fibroblasts. FREO was significantly anti-proliferative to these cells. FREO significantly inhibited the production of collagen III, IP-10, and ICAM-1. Genome-wide gene expression analysis showed that FREO modulated global gene expression. It also robustly affected signaling pathways which are relevant to inflammation and tissue remodeling.

## Conflicts of interest

X.H., D.R., and T.P. are employees of dōTERRA, where the study agent FREO was manufactured.

## Footnotes

1 FREO, frankincense essential oil.

2 SRB, sulforhodamine B.

3 IPA, Ingenuity^®^ Pathway Analysis.

4 IP-10, interferon gamma-induced protein 10.

5 ICAM-1, intracellular cell adhesion molecule 1.
