# Supplementary material for: Biological activities of frankincense essential oil in human dermal fibroblasts
Source: Biochim Open. 2017 Feb 3;4:31–5. doi: 10.1016/j.biopen.2017.01.003 (PMC5801908; doi:10.1016/j.biopen.2017.01.003)

Table S1. Glossary of the Biomarkers of System HDF3CGF Used in the Study

| **Readout** | **Description** |
| --- | --- |
| **CCL2/MCP-1** | MCP-1 system is a chemokine that mediates recruitment of monocytes and T cells into sites of inflammation. MCP-1 is categorized as an inflammation-related activity in the HDF3CGF system modeling Th1 inflammation involved in wound healing and matrix remodeling. |
| **CD106/VCAM-1** | VCAM-1 is a cell adhesion molecule that mediates adhesion of monocytes and T cells to endothelial cells. VCAM-1 is categorized as an inflammation-related activity. |
| **CD54/ICAM-1** | ICAM-1 is a cell adhesion molecule that mediates leukocyte-endothelial cell adhesion and leukocyte recruitment. ICAM-1 is categorized as an inflammation-related activity. |
| **Collagen I** | Collagen I is involved in tissue remodeling and fibrosis, and is the most common fibrillar collagen that is found in skin, bone, tendons and other connective tissues. Collagen I is categorized as a tissue remodeling-related activity. |
| **Collagen III** | Collagen III is an extracellular matrix protein and fibrillar collagen found in extensible connective tissues (skin, lung and vascular system) and is involved in cell adhesion, cell migration, tissue remodeling. Collagen III is categorized as a tissue remodeling-related activity. |
| **CXCL10/IP-10** | IP-10 is a chemokine that mediates T cell, monocyte and dendritic cell chemotaxis. IP-10 is categorized as an inflammation-related activity. |
| **CXCL11/I-TAC** | I-TAC is a chemokine that mediates T cell and monocyte chemotaxis. I-TAC is categorized as an inflammation-related activity. |
| **CXCL8/IL-8** | IL-8 is a chemokine that mediates neutrophil recruitment into acute inflammatory sites. IL-8 is categorized as an inflammation-related activity. |
| **CXCL9/MIG** | MIG is a chemokine that mediates T cell recruitment. MIG is categorized as an inflammation-related activity. |
| **EGFR** | EGFR is a cell surface receptor for epidermal growth factor involved in cell proliferation during development as well as tumor growth. EGFR is involved in Epithelial cell proliferation, epithelial cell differentiation keratinocyte proliferation, tissue remodeling. EGFR is categorized as a tissue remodeling-related activity. |
| **M-CSF** | M-CSF is a secreted and cell surface cytokine that mediates macrophage differentiation. M-CSF is categorized as a tissue remodeling-related activity. |
| **MMP-1** | MMP-1 is an interstitial collagenase that degrades collagens I, II and III and is involved in the process of tissue remodeling. MMP-1 is categorized as a tissue remodeling-related activity. |
| **PAI-I** | PAI-I is a serine proteinase inhibitor and inhibitor of tissue plasminogen activator (tPA) and urokinase (uPA) and is involved in tissue remodeling and fibrinolysis. PAI-I is categorized as a tissue remodeling-related activity. |
| **Proliferation_72hr** | Proliferation_72hr in the HDF3CGF system is a measure of dermal fibroblast proliferation which is important to the process of wound healing and fibrosis. |
| **SRB** | SRB is a measure of the total protein content of dermal fibroblasts. Cell viability of adherent cells is measured by Sulforhodamine B (SRB) staining, a method that determines cell density by measuring total protein content of test wells. |
| **TIMP-1** | TIMP-1 is a tissue inhibitor of matrix metalloprotease-7 (MMP-7) and other MMPs, and is involved in tissue remodeling, angiogenesis and fibrosis. TIMP-1 is categorized as a tissue remodeling-related activity. |
| **TIMP-2** | TIMP-2 is a tissue inhibitor of matrix metalloproteases and is involved in tissue remodeling, angiogenesis and fibrosis. TIMP-2 is categorized as a tissue remodeling-related activity. |

Table S2. Top 83 genes modulated by FREO (fold change in log_2_ (relative ratio) form)

| **Illumina Gene ID** | **Fold Change** | **Definition** |
| --- | --- | --- |
| HLA-A | 2.50 | Homo sapiens major histocompatibility complex, class I, A (HLA-A), mRNA. |
| UMODL1 | 2.08 | Homo sapiens uromodulin-like 1 (UMODL1), transcript variant 2, mRNA. |
| TFRC | 1.98 | Homo sapiens transferrin receptor (p90, CD71) (TFRC), mRNA. |
| HSPE1 | 1.86 | Homo sapiens heat shock 10kDa protein 1 (chaperonin 10) (HSPE1), mRNA. |
| LOC100131733 | 1.83 | PREDICTED: Homo sapiens hypothetical LOC100131733 (LOC100131733), mRNA. |
| LOC441743 | 1.82 | Homo sapiens similar to C367G8.3 (novel protein similar to RPL23A (60S ribosomal protein L23A)) (LOC441743), mRNA. |
| HNRPH1 | 1.79 | Homo sapiens heterogeneous nuclear ribonucleoprotein H1 (H) (HNRPH1), mRNA. |
| MGLL | 1.72 | Homo sapiens monoglyceride lipase (MGLL), transcript variant 1, mRNA. |
| LOC645638 | 1.69 | PREDICTED: Homo sapiens misc_RNA (LOC645638), miscRNA. |
| ZSWIM2 | 1.68 | Homo sapiens zinc finger, SWIM-type containing 2 (ZSWIM2), mRNA. |
| LRRC37A2 | 1.67 | Homo sapiens leucine rich repeat containing 37, member A2 (LRRC37A2), mRNA. |
| EEF1B2 | 1.67 | Homo sapiens eukaryotic translation elongation factor 1 beta 2 (EEF1B2), transcript variant 2, mRNA. |
| NR4A3 | 1.63 | Homo sapiens nuclear receptor subfamily 4, group A, member 3 (NR4A3), transcript variant 3, mRNA. |
| SCARNA14 | 1.63 | Homo sapiens small Cajal body-specific RNA 14 (SCARNA14), guide RNA. |
| TSC22D1 | 1.62 | Homo sapiens TSC22 domain family, member 1 (TSC22D1), transcript variant 2, mRNA. |
| MAP2K3 | 1.60 | Homo sapiens mitogen-activated protein kinase kinase 3 (MAP2K3), transcript variant A, mRNA. |
| FABP3 | 1.60 | Homo sapiens fatty acid binding protein 3, muscle and heart (mammary-derived growth inhibitor) (FABP3), mRNA. |
| LOC644466 | 1.60 | PREDICTED: Homo sapiens hypothetical protein LOC644466 (LOC644466), mRNA. |
| MGC87895 | 1.60 | PREDICTED: Homo sapiens similar to ribosomal protein S14 (MGC87895), mRNA. |
| HS.378070 | 1.59 | hn28d09.x1 NCI_CGAP_Thy7 Homo sapiens cDNA clone IMAGE:3023441 3, mRNA sequence |
| C5ORF4 | 1.58 | Homo sapiens chromosome 5 open reading frame 4 (C5orf4), mRNA. |
| LOC100132515 | 1.57 | PREDICTED: Homo sapiens similar to hCG2045429 (LOC100132515), mRNA. |
| BTN3A2 | 1.57 | Homo sapiens butyrophilin, subfamily 3, member A2 (BTN3A2), mRNA. |
| LOC653994 | 1.56 | PREDICTED: Homo sapiens similar to Eukaryotic translation initiation factor 4H (eIF-4H) (Williams-Beuren syndrome chromosome region 1 protein homolog), transcript variant 2 (LOC653994), mRNA. |
| DBN1 | 1.56 | Homo sapiens drebrin 1 (DBN1), transcript variant 1, mRNA. |
| WDR17 | 1.55 | Homo sapiens WD repeat domain 17 (WDR17), transcript variant 1, mRNA. |
| HS.128393 | 1.55 | BX092776 NCI_CGAP_GC4 Homo sapiens cDNA clone IMAGp998E034025 ; IMAGE:1587818, mRNA sequence |
| LOC93556 | 1.55 | PREDICTED: Homo sapiens hypothetical protein BC011266, transcript variant 3 (LOC93556), mRNA. |
| LOC100130562 | 1.55 | PREDICTED: Homo sapiens hypothetical protein LOC100130562, transcript variant 1 (LOC100130562), mRNA. |
| MBL1P1 | 1.55 | Homo sapiens mannose-binding lectin (protein A) 1, pseudogene 1 (MBL1P1) on chromosome 10. |
| MIR302B | 1.55 | Homo sapiens microRNA 302b (MIR302B), microRNA. |
| LOC100130289 | 1.55 | PREDICTED: Homo sapiens misc_RNA (LOC100130289), miscRNA. |
| S100A4 | 1.53 | Homo sapiens S100 calcium binding protein A4 (S100A4), transcript variant 2, mRNA. |
| OR2M1P | 1.53 | Homo sapiens olfactory receptor, family 2, subfamily M, member 1 pseudogene (OR2M1P), non-coding RNA. |
| BZW1 | 1.53 | Homo sapiens basic leucine zipper and W2 domains 1 (BZW1), mRNA. XM_943165 |
| OR4A47 | 1.53 | Homo sapiens olfactory receptor, family 4, subfamily A, member 47 (OR4A47), mRNA. |
| CGGBP1 | 1.52 | Homo sapiens CGG triplet repeat binding protein 1 (CGGBP1), transcript variant 1, mRNA. |
| LPPR5 | 1.51 | Homo sapiens lipid phosphate phosphatase-related protein type 5 (LPPR5), transcript variant 1, mRNA. |
| LOC100131980 | 1.51 | PREDICTED: Homo sapiens similar to zinc finger protein 705A (LOC100131980), mRNA. |
| HNRNPA1 | 1.50 | Homo sapiens heterogeneous nuclear ribonucleoprotein A1 (HNRNPA1), transcript variant 2, mRNA. |
| LOC645231 | 1.50 | PREDICTED: Homo sapiens misc_RNA (LOC645231), miscRNA. |
| KRTAP5-5 | 1.50 | Homo sapiens keratin associated protein 5-5 (KRTAP5-5), mRNA. |
| C13ORF33 | -1.50 | Homo sapiens chromosome 13 open reading frame 33 (C13orf33), mRNA. |
| HS.505364 | -1.50 | Homo sapiens cDNA FLJ13402 fis, clone PLACE1001456 |
| KLHDC8A | -1.50 | Homo sapiens kelch domain containing 8A (KLHDC8A), mRNA. |
| SUDS3 | -1.50 | Homo sapiens suppressor of defective silencing 3 homolog (S. cerevisiae) (SUDS3), mRNA. |
| LOC100133923 | -1.50 | PREDICTED: Homo sapiens hypothetical protein LOC100133923 (LOC100133923), mRNA. |
| FLJ42709 | -1.50 | PREDICTED: Homo sapiens hypothetical gene supported by AK124699 (FLJ42709), mRNA. |
| ORM2 | -1.51 | Homo sapiens orosomucoid 2 (ORM2), mRNA. |
| EIF2AK3 | -1.51 | Homo sapiens eukaryotic translation initiation factor 2-alpha kinase 3 (EIF2AK3), mRNA. |
| TNFSF12-TNFSF13 | -1.51 | Homo sapiens TNFSF12-TNFSF13 readthrough (TNFSF12-TNFSF13), mRNA. |
| RBMY1A3P | -1.51 | Homo sapiens RNA binding motif protein, Y-linked, family 1, member A3 pseudogene (RBMY1A3P), non-coding RNA. |
| MIR25 | -1.52 | Homo sapiens microRNA 25 (MIR25), microRNA. |
| SFTPA2B | -1.52 | Homo sapiens surfactant protein A2B (SFTPA2B), mRNA. XM_001133043 XM_001133049 XM_001133054 |
| IGSF6 | -1.53 | Homo sapiens immunoglobulin superfamily, member 6 (IGSF6), mRNA. |
| ZCCHC2 | -1.53 | Homo sapiens zinc finger, CCHC domain containing 2 (ZCCHC2), mRNA. |
| TXNRD1 | -1.53 | Homo sapiens thioredoxin reductase 1 (TXNRD1), transcript variant 5, mRNA. |
| LOC340357 | -1.53 | Homo sapiens hypothetical LOC340357 (LOC340357), non-coding RNA. |
| FUT8 | -1.53 | Homo sapiens fucosyltransferase 8 (alpha (1,6) fucosyltransferase) (FUT8), transcript variant 3, mRNA. |
| CSMD1 | -1.54 | Homo sapiens CUB and Sushi multiple domains 1 (CSMD1), mRNA. |
| AVPR1B | -1.54 | Homo sapiens arginine vasopressin receptor 1B (AVPR1B), mRNA. |
| USP18 | -1.55 | Homo sapiens ubiquitin specific peptidase 18 (USP18), mRNA. |
| MED27 | -1.55 | Homo sapiens mediator complex subunit 27 (MED27), mRNA. |
| CCNB3 | -1.55 | Homo sapiens cyclin B3 (CCNB3), transcript variant 3, mRNA. |
| TP53INP2 | -1.56 | Homo sapiens tumor protein p53 inducible nuclear protein 2 (TP53INP2), mRNA. |
| TP53INP1 | -1.56 | Homo sapiens tumor protein p53 inducible nuclear protein 1 (TP53INP1), mRNA. |
| CD97 | -1.57 | Homo sapiens CD97 molecule (CD97), transcript variant 1, mRNA. |
| KCTD1 | -1.57 | Homo sapiens potassium channel tetramerisation domain containing 1 (KCTD1), mRNA. |
| MMP9 | -1.61 | Homo sapiens matrix metallopeptidase 9 (gelatinase B, 92kDa gelatinase, 92kDa type IV collagenase) (MMP9), mRNA. |
| ITPRIPL2 | -1.61 | Homo sapiens inositol 1,4,5-triphosphate receptor interacting protein-like 2 (ITPRIPL2), mRNA. |
| CCL5 | -1.61 | Homo sapiens chemokine (C-C motif) ligand 5 (CCL5), mRNA. |
| BAHCC1 | -1.63 | Homo sapiens BAH domain and coiled-coil containing 1 (BAHCC1), mRNA. |
| LOC653160 | -1.63 | PREDICTED: Homo sapiens Hypothetical protein LOC653160, transcript variant 2 (LOC653160), mRNA. |
| DMPK | -1.64 | Homo sapiens dystrophia myotonica-protein kinase (DMPK), transcript variant 1, mRNA. |
| MX2 | -1.65 | Homo sapiens myxovirus (influenza virus) resistance 2 (mouse) (MX2), mRNA. |
| PRKAG2 | -1.68 | Homo sapiens protein kinase, AMP-activated, gamma 2 non-catalytic subunit (PRKAG2), transcript variant a, mRNA. |
| LOC730249 | -1.69 | PREDICTED: Homo sapiens similar to Immune-responsive protein 1 (LOC730249), mRNA. |
| MMP12 | -1.70 | Homo sapiens matrix metallopeptidase 12 (macrophage elastase) (MMP12), mRNA. |
| ESM1 | -1.75 | Homo sapiens endothelial cell-specific molecule 1 (ESM1), mRNA. |
| MYH11 | -1.85 | Homo sapiens myosin, heavy chain 11, smooth muscle (MYH11), transcript variant SM1A, mRNA. |
| CFB | -1.86 | Homo sapiens complement factor B (CFB), mRNA. |
| CCL5 | -1.98 | Homo sapiens chemokine (C-C motif) ligand 5 (CCL5), mRNA. |
| CYP26B1 | -2.19 | Homo sapiens cytochrome P450, family 26, subfamily B, polypeptide 1 (CYP26B1), mRNA. |

Table S3. Top 20 genes regulated by FREO in the canonical Hepatic Fibrosis/Hepatic Stellate Cell Activation pathway. Fold change over vehicle was shown in log_2_ ratio form.


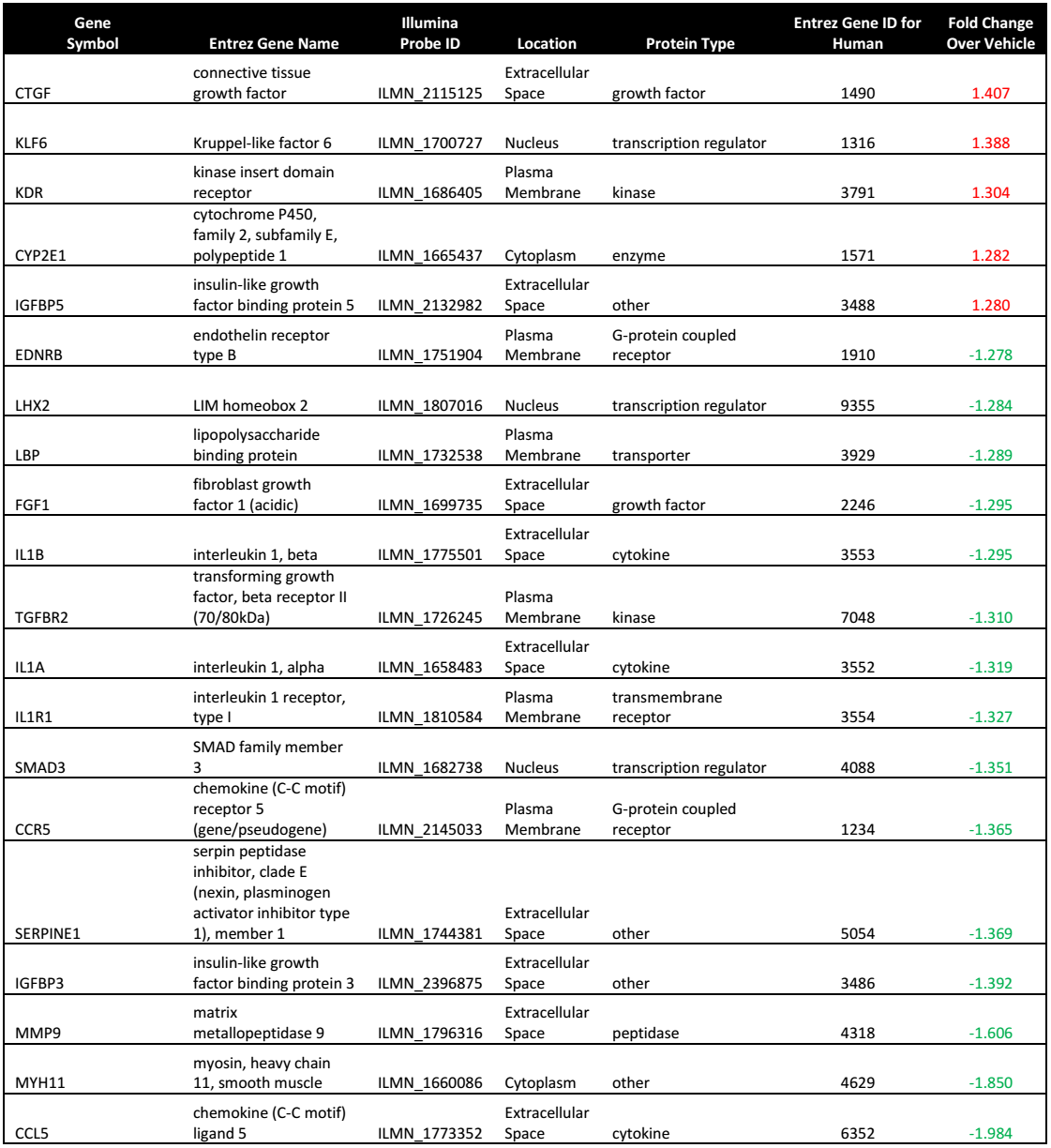


Table S4. Top 15 genes regulated by FREO in the canonical communication between innate and adaptive immune cells pathway. Fold change over vehicle was shown in log_2_ ratio form.


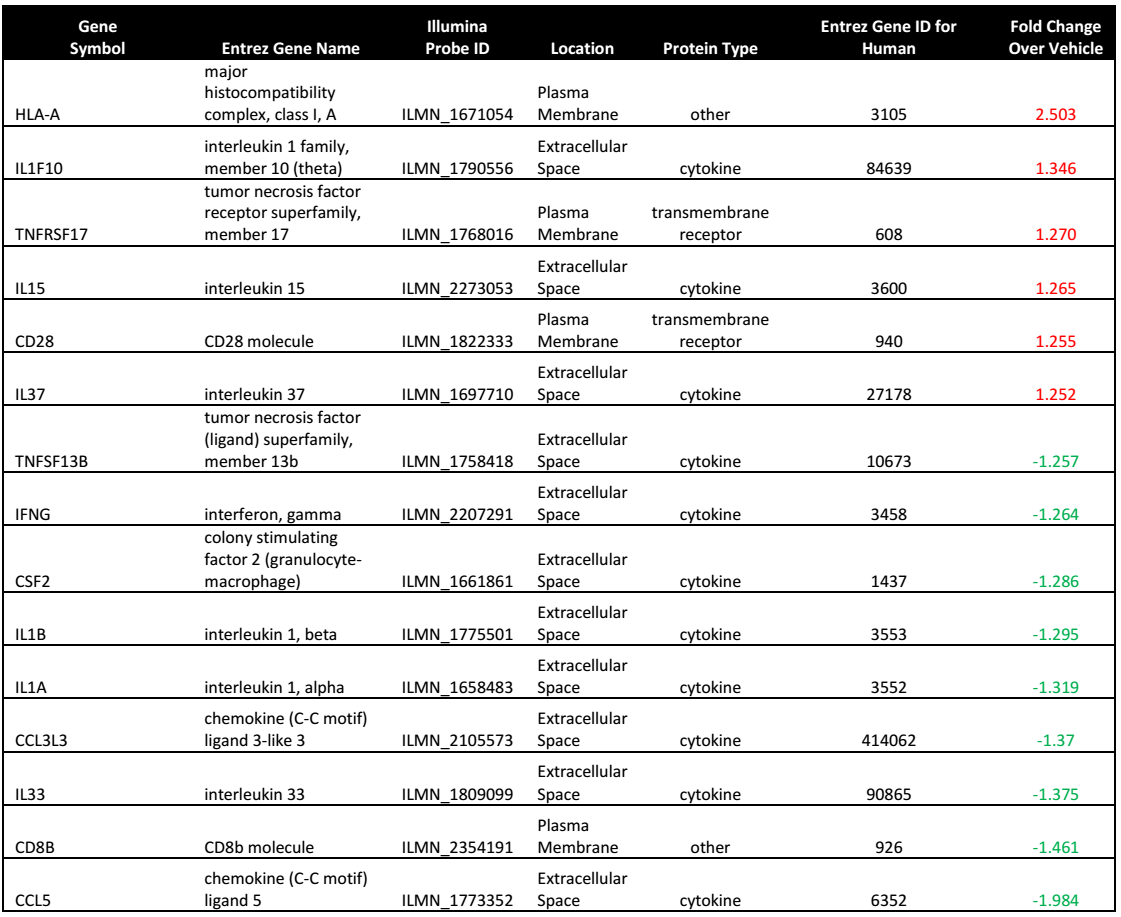


Table S5. Top 20 genes regulated by FREO in the canonical agranulocyte adhesion and diapedesis pathway. Fold change over vehicle was shown in log_2_ ratio form.


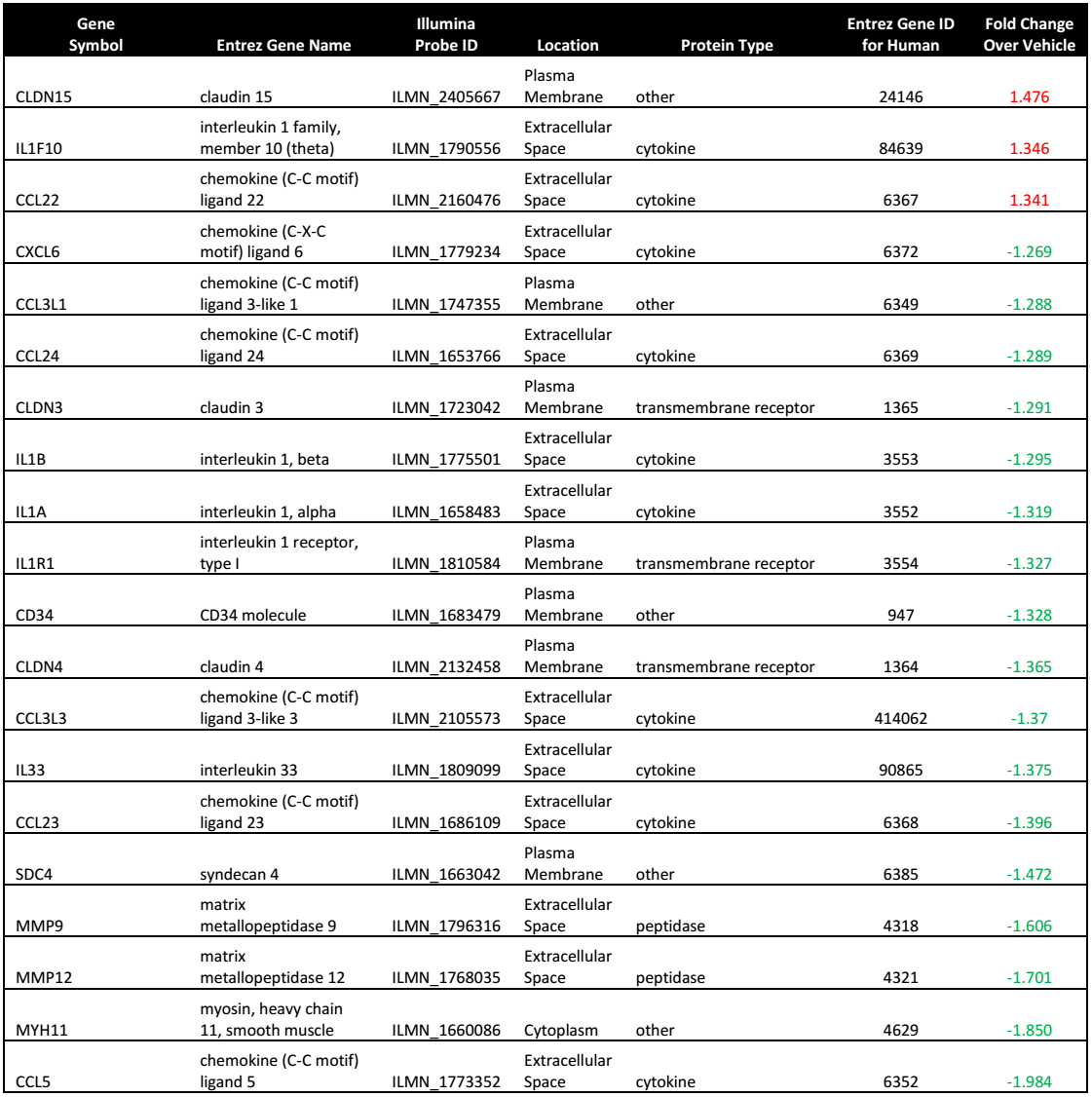


Table S6. Top 10 genes regulated by FREO in the canonical neuroprotective role of THOP1 in Alzheimer’s disease pathway. Fold change over vehicle was shown in log_2_ ratio form.


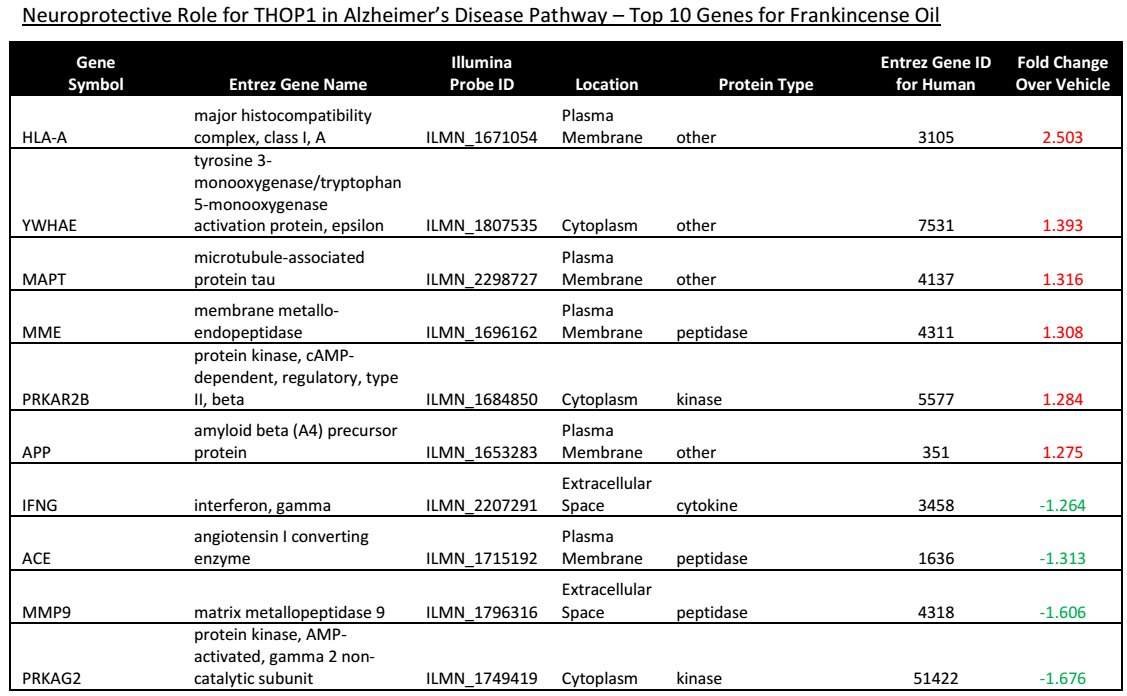

Supplement: Supplementary file 1 [file mmc1.docx]
